# Effectiveness of an Educational and Counseling Program (the Green Mother Project Phase 2) to Enhance Breastfeeding and Improve Mothers’ Diets From an Environmental Perspective: Protocol for a Cluster Randomized Controlled Trial

**DOI:** 10.2196/80358

**Published:** 2026-01-26

**Authors:** Rosa Maria Cabedo-Ferreiro, Liudmila Liutsko, Judit Cos-Busquets, Rosa García-Sierra, Margalida Colldeforns-Vidal, Azahara Reyes-Lacalle, Pere Torán-Monserrat, M Mercedes Vicente-Hernández, Miriam Gómez-Masvidal, Concepció Violán, Laura Montero-Pons, Gemma Falguera-Puig, Gemma Cazorla-Ortiz

**Affiliations:** 1 Atenció a la Salut Sexual I Reproductiva (ASSIR) Granollers Institut Català de la Salut Granollers Spain; 2 (2021-SGR-793) Consolidated Research Group on Sexual and Reproductive Healthcare (GRASSIR) Barcelona Spain; 3 Unitat de Suport a la Recerca Metropolitana Nord Institut Universitari d'Investigació en Atenció Primària Jordi Gol Mataró Spain; 4 Dep. Health Sciences; Group PSICONLINE Universidad Internacional De La Rioja Logroño Spain; 5 Atenció a la Salut Sexual I Reproductiva (ASSIR) Sabadell Institut Català de la Salut Sabadell Spain; 6 (2021-SGR-0148) Multidisciplinary Research Group in Health and Society (GREMSAS) Barcelona Spain; 7 Institut d’Investigació Germans Trias i Pujol (IGTP) Badalona Spain; 8 Department of Medicine, Faculty of Medicine Universitat de Girona Girona Spain; 9 Atenció a la Salut Sexual I Reproductiva (ASSIR) Badalona - Sant Adrià Institut Català de la Salut Sant Adrià del Besós Spain; 10 Atenció a la Salut Sexual I Reproductiva (ASSIR) Mataró Institut Català de la Salut Mataró Spain; 11 Universitat Autònoma de Barcelona Cerdanyola del Vallès Spain; 12 Institut Universitari D'Investigació en Atenció Primària Jordi Gol (IDIAPJGol) (2021 SGR 01537) Grup de Recerca en Impacte de les Malalties Cròniques i les seves Trajectòries (GRIMTra) Barcelona Spain; 13 Institut Universitari D'Investigació en Atenció Primària Jordi Gol (IDIAPJGol) (RD21/0016/0029) El grupo de investigación en servicios sanitarios en Atención Primaria (GrenSSAP), RICAPPS Barcelona Spain

**Keywords:** public health, planetary health, nutrition, post partum, breastfeeding, bottle feeding, counseling, prenatal education, health education, public health education for professionals

## Abstract

**Background:**

Exclusive breastfeeding is recommended as healthier and more sustainable than formula feeding. It produces less waste, requires fewer resources, and has a smaller environmental impact. Breastfeeding has some environmental impact related to increased maternal dietary needs and the use of feeding accessories. In light of the global climate emergency and suboptimal breastfeeding rates, targeted interventions are urgently needed to promote sustainable infant feeding practices. There are few studies that evaluate sustainability interventions in the postpartum period.

**Objective:**

The objective of this study is to evaluate the effectiveness of an educational and counseling intervention on breastfeeding and healthy maternal nutrition from an environmental perspective.

**Methods:**

A multicenter prospective intervention study is being conducted in 2 cohorts in primary care centers and hospitals in the north metropolitan area of Barcelona. The control group received standard obstetric care. The experimental group additionally received an educational intervention and health care support on breastfeeding and healthy and sustainable maternal nutrition. Pregnant women were monitored from 24 weeks of gestation to 6 months post partum. The rates of different types of breastfeeding, the women’s diet, and the associated environmental impacts (climate change and water footprint) will be analyzed to assess the effectiveness of the intervention.

**Results:**

The development of the educational and counseling intervention has been completed, including the creation of the *Guide to Good Practices in Breastfeeding, Nutrition, and Sustainability*. Health care professionals received targeted training. Recruitment of pregnant women was conducted from December 2023 to December 2024. Prenatal education sessions and specialized care pathways were designed and implemented. Breastfeeding-friendly spaces were adapted to support the participating centers. Data collection for monitoring breastfeeding practices, maternal diet, and environmental impact indicators (carbon footprint and water footprint), with the follow-up period of 6 months post partum, was extended until September 2025, with a complementary missing data collection in October 2025. Data cleaning for final analysis is expected to conclude by January 2026. This study hypothesizes that mothers who receive higher levels of education and counseling support will (1) breastfeed for a longer duration, (2) adopt healthier and more sustainable dietary practices, and (3) reduce environmental impacts associated with both infant feeding accessories and dietary choices.

**Conclusions:**

We expect an increase in the incidence and prevalence rates of breastfeeding and a shift toward a healthy and sustainable diet with low environmental impact.

**Trial Registration:**

ClinicalTrials.gov NCT05729581; https://clinicaltrials.gov/study/NCT05729581

**International Registered Report Identifier (IRRID):**

DERR1-10.2196/80358

## Introduction

The World Health Organization (WHO) recommends exclusive breastfeeding (EBF) for the first 6 months of life, continuing until the child is aged 2 years or as desired by mother and child, as it provides numerous physical and mental health benefits [1-5]. Factors that impact breastfeeding include the type of delivery; the socioeconomic status of the mother; her return to work; support from partners, families, and peer groups; and the level of prenatal breastfeeding education [6]. Breastfeeding is a sustainable and environmentally friendly infant feeding method compared to formula feeding, as it generates less waste and produces minimal greenhouse gas (GHG) emissions; therefore, it has a smaller carbon footprint. However, we cannot affirm that breastfeeding has a 0 cost from an environmental point of view because the impact of the increase in maternal diet and the use of breastfeeding accessories must be calculated [1,7-9]. Maternal diet [10,11] and associated consumption habits (local purchasing; type of energy and cooking used) can also contribute not only to the quality of nutrition [12] but also to sustainability and environmental protection [13,14]. The Paris Agreement has set ambitious targets for Europe to reduce its carbon footprint, aiming for a 55% reduction in GHG emissions by 2030 and climate neutrality by 2050 [15]. Food production accounts for 30% of the total GHG emissions. A healthy diet based on the consumption of vegetables, legumes, and low-impact foods contributes to reducing the carbon footprint [8]. By contrast, the manufacturing and distribution of industrial infant formulas harm the environment, creating polluting waste and requiring substantial use of energy and water [15].

Supporting breastfeeding mothers is crucial to achieving the WHO 2025 target (reaching 50% EBF during the first 6 months of life) [16] and the goals of the United Nations Paris Agreement [15]. However, despite many mothers’ desire to breastfeed, obstacles and difficulties often lead to premature cessation of breastfeeding [1,17].

Health professionals play a vital role in providing support and guidance for successful and prolonged breastfeeding. Specific training for professionals followed by advice and education for users has been shown to be effective in improving breastfeeding care [18]. Shafaei et al [19], in 2020, demonstrated that after a bad experience with breastfeeding, prenatal education or counseling solves the main breastfeeding problems and improves breastfeeding self-efficacy. The implementation of supportive interventions in both the prenatal and postpartum periods can improve the rate, exclusivity, and duration of breastfeeding [19-21]. Souza et al [20] confirmed the effectiveness of an intervention also in the postpartum period. Educational programs have demonstrated greater effectiveness than support programs conducted through telephone and promotional materials, such as leaflets [22]. Sattari et al [22], in a systematic review of 30 trials, could not determine whether the combination of education with support was more effective than education alone. If we consider the information on a healthy diet, a Cochrane review with 1090 women evaluated the effectiveness in terms of perinatal outcomes of prenatal dietary education [23]. Several studies have demonstrated the effectiveness of educational interventions and professional support in improving both the rates and duration of breastfeeding [18-20]. However, existing research has predominantly focused on clinical and psychosocial aspects, with limited attention given to the interrelationship among health education, sustainable nutrition, and environmental impact.

The education of health care professionals is a key factor. Previous studies have identified a lack of environmental knowledge among health professionals, which limits their ability to guide families toward more sustainable practices [24]. Jadotte et al [24] emphasized the need to incorporate environmental content into health care education so that professionals are equipped to transfer this knowledge to the wider population.

Research into educational interventions on the environmental benefits of breastfeeding and mothers’ diets is essential to achieve changes in the attitude toward prolonged breastfeeding and a healthy and sustainable diet for the whole family. Currently, there are no studies on the impact of a training intervention for professionals and education for users on breastfeeding and a healthy and sustainable diet. Therefore, conducting research in this area, increasing the knowledge of professionals, and updating standard prenatal courses can contribute to raising awareness and promoting a change in attitude toward the protection of the environment and the promotion of public health as well as ensuring the future well-being of both the population and the planet [25,26].

The main objective of our study is to evaluate the effectiveness of an educational intervention with counseling and material resources for breastfeeding in improving breastfeeding rates, healthy diet, and environmental impacts. At the same time, it is hoped that the rates of different types of breastfeeding up to 6 months of life of the newborn can be calculated; the quality of the maternal diet in terms of calories and macro- and micronutrients can be described; and, finally, the environmental impact of infant and maternal nutrition between the 2 groups can be compared. To sum up, this study aims to evaluate whether a nutritional and environmental educational intervention improves EBF rates and maternal diet quality and reduces environmental impact compared to standard obstetric care.

We hypothesize that women who receive a nutritional and environmental educational intervention will have higher rates of EBF and a more balanced diet than women who receive standard obstetric care. Furthermore, the environmental impact produced by the participants who receive intervention will be smaller than that produced by the women who receive the standard obstetric care.

## Methods

This research protocol is part of a broader project, where in phase 1, the environmental impact of infant feeding is calculated, considering the accessories necessary for it and the maternal diet, accounting for food consumption habits [9,12,27]. With these results, it has been possible to design the informative materials and courses that were used in the intervention, that is, phase 2 of the Green Mother project.

### Design of This Study

A multicenter intervention cluster study with a randomized controlled trial is being conducted in the north metropolitan area of Barcelona, Spain, between 2023 and 2026. It is implemented in the public universal primary care system, specifically from the reproductive and sexual health care units (ASSIRs; Catalan: Atenció a la Salut Sexual i Reproductiva) [28,29] and in the territory’s reference public hospitals for childbirth care. The participating centers were randomly assigned (primary care and their reference hospitals).

### Participants and Inclusion and Exclusion Criteria

The study population is composed of pregnant women from the ASSIR centers, where pregnancy monitoring is conducted, with deliveries planned in the reference hospitals. Participants were enrolled in order of care, provided they met the inclusion criteria and had been accepted into the study, until the predetermined sample size was reached. The inclusion criteria were as follows: (1) receiving care in these ASSIRs and hospitals; (2) being aged 16 years or more, with a gestational age between 24 and 32 weeks; and (3) wishing to breastfeed. The exclusion criteria were as follows: (1) linguistic barriers that prevented the understanding of the information and the ability to complete the questionnaires and (2) not wishing to participate. Participants were followed from 24 to 32 weeks of gestation to 6 months post partum. The follow-up period lasted until September 2025.

### Ethical Considerations

We followed the guidelines of the Declaration of Helsinki regarding bioethical principles of clinical research. This research protocol was approved by the research ethics committee of the Jordi Gol Primary Care Research Institute as well as the reference hospitals participating in this study: Hospital Parc Taulí, Hospital General de Granollers, Hospital Germans Trias i Pujol, and Hospital de Mataró. The final version of this protocol (22/101-P) was approved and signed on February 22, 2023.

Data collected through questionnaires on REDCap (Research Electronic Data Capture; Vanderbilt University) are encrypted and stored in a secure database. Data for the study were collected directly from project participants based on their prior written consent, in accordance with the provisions of Articles 6.1.a and 9.2.a of the General Data Protection Regulation.

### Sample Size Calculation

A total of 126 participants is needed in each group to detect a statistically significant difference between the 2, assuming a confidence level of 95% and a power of 80% in a 2-tailed test, according to the calculations of the GRANMO sample size and power calculator (version 8.0; Datarus). Based on the data published in 2019 on the population studied, the EBF rate at 6 months would be 17% in the control group (CG) and 34% in the experimental group (EG) [21]. The loss to follow-up rate is estimated to be 20%. A total of 252 participants will be studied, with 126 (50%) in each group.

### Procedure

#### Recruitment

Midwives at the participating centers invited pregnant women who met the inclusion criteria and provided consent to take part in this study by signing the informed consent form.

#### Intervention

Randomization was performed at the cluster level (health care centers), ensuring that all participants within a given center received the same type of care. The intervention study included two groups: (1) CG, which received standard obstetric care (ASSIRs of Badalona, Sant Adrià, and Mataró together with Hospital Germans Trias i Pujol and Hospital de Mataró) and (2) EG or intervention group, which received an extra educational program and health care support (ASSIRs of Granollers, Sabadell, and Cerdanyola with Hospital Parc Taulí and Hospital General de Granollers).

A multimodal intervention included activities for professionals and the study participants, such as dissemination, training, and counseling support actions.

**Figure 1:**
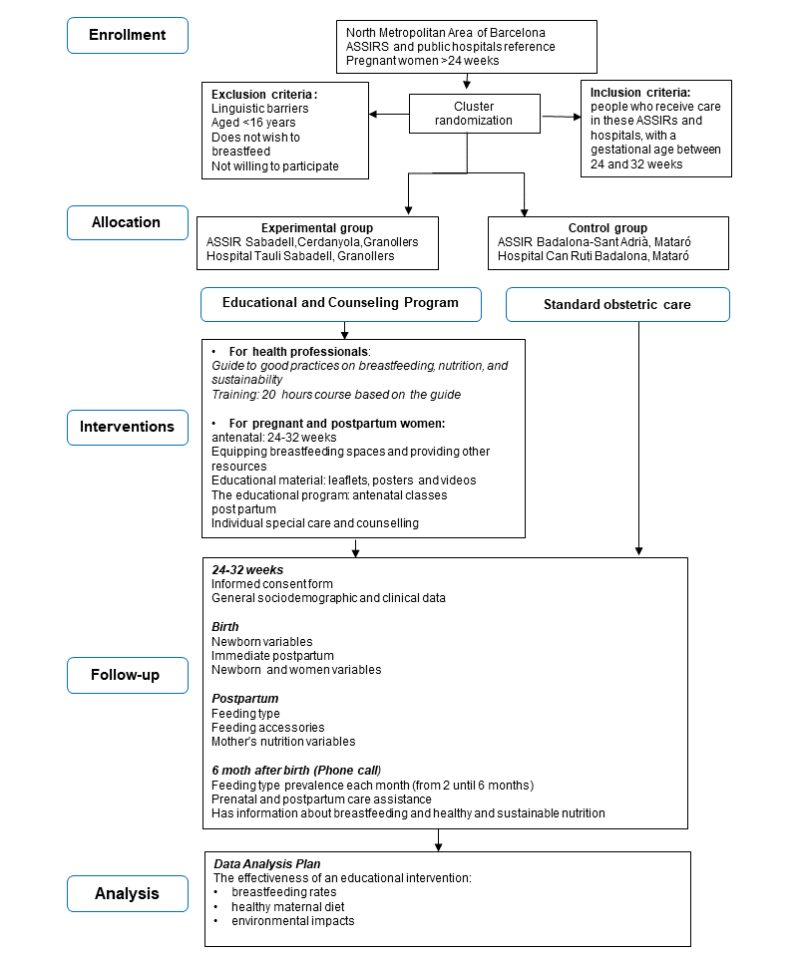
Workflow chart.

The dimensions of the intervention (Table 1 and Figure 1) are mentioned subsequently.

**Table 1:** The educational and support intervention program overview performed in the experimental group centers.

Intervention target and intervention type (dimensions)			Before recruitment (primary care)		Pregnancy				Delivery		Postpartum visits										
					24 gestational weeks		28-36 gestational weeks		Birth		Immediate		Early		Month 1		Month 3		Month 4		Month 6
					Primary care				Hospital				Primary care								
**Health professionals**																					
	Creation of the Guide to Good Practices in Breastfeeding, Nutrition, and Sustainability	✓																			
	Training of health workers (virtual course: 20 h)	✓																			
**Pregnant and postpartum persons**																					
	Equipping breastfeeding spaces and providing other resources	✓																			
	Educational leaflets, posters with QR codes, and videos	✓																			
	The educational program: special sessions on sustainable feeding and nutrition for pregnant persons: (1) breastfeeding, (2) sustainable feeding, and (3) healthy nutrition (1 hour per session^a^)					✓															
	Individual special care and counseling for postpartum persons in hospitals and primary care centers following the guide			✓				✓		✓		✓		✓		✓				✓	

^a^Versus standard educational courses and standard obstetric care in the control group.

For primary care and hospital health professionals (with the aim of enhancing and homogenizing knowledge), the first dimension was the creation of a *Guide to Good Practices in Breastfeeding, Nutrition, and Sustainability* (based on breastfeeding topics in the *Clinical Practice Guide on Breastfeeding* published by the Spanish Ministry of Health). The content was structured around the following topics: anatomy, physiology, benefits, initiation, monitoring, and challenges of breastfeeding; healthy maternal nutrition; sustainability; and sustainable diets. It was used in both primary care centers and hospitals to achieve excellence in care for mothers and babies and included updated information on breastfeeding and healthy and sustainable nutrition.

The second dimension was training provided to health professionals, such as midwives, pediatric nurses, and pediatricians, in ASSIR centers, primary care centers, and hospitals. This was conducted through a 20-hour course taught by the Green Mother research team using a virtual training platform. The course consisted of 12 topics based on the content of the guide named in the first dimension and the results of phase 1 of the Green Mother study on breastfeeding, maternal diet, and environmental impact. Viewing of the videos was monitored, and participants completed an assessment consisting of a series of questions on the content to evaluate their knowledge. These courses were scheduled between 2023 and 2024, before the start of participant recruitment for the study.

For pregnant and postpartum women, the third dimension was equipment for waiting rooms, other spaces, and resources. Financial resources were allocated to support the adaptation of primary care centers and hospitals to provide optimal breastfeeding counseling, for example, creating cozy and calm spaces with sofas and supports (ie, nursing pillows, breast pumps, nipple shields, and reusable nursing pads).

The fourth dimension included educational materials. Information brochures were created for participants with advice on effective breastfeeding; tips or information messages for a healthy and sustainable diet; as well as details on the environmental impacts of formula milk, accessories, and food.

Informative videos on breastfeeding and healthy and sustainable nutrition, developed in accordance with the guidelines, were displayed in the waiting areas of primary care centers and hospitals.

Posters for surgeries and waiting rooms with QR codes were provided, where all the project information could be accessed.

The fifth dimension, the prenatal education course taught by trained professionals, was expanded and adapted with specific information on the Green Mother project. It included 3 sessions, namely breastfeeding, healthy maternal diet, and sustainability. Presentations in Microsoft PowerPoint format and recorded classes on healthy eating, breastfeeding, and environmental education were designed for users who cannot attend classes. Each class lasted 1 hour.

The sixth dimension was specific care for participants from recruitment to the study until post partum. Counseling was carried out at an individual level, both in primary care centers and at hospital admission, following the *Guide to Good Practices in Breastfeeding, Nutrition, and Sustainability*(first dimension) and offering support for behavioral changes that facilitate EBF and a healthier and more sustainable diet using the resources provided.

### Data Collection and Data Sources

All data were collected through questionnaires completed by the researcher in charge of participant recruitment, using the REDCap secure web application [30] housed in the Catalan Health Institute.

A range of variables were collected to enable the computation of outcome measures used to evaluate the effectiveness of the intervention (refer to the Data Analysis Plan section).

The timing of participants’ data collection is presented in Table 2, and the variables (more details are provided in Multimedia Appendix 1) are presented in Textbox 1.

Data collection by time, place, and the instruments used are presented in Table 2.

**Table 2:** Data collection protocol for the Green Mother phase 2 study.

Validated instrument			Primary care		Hospital				Primary care					Phone call				
			24 to 32 weeks of pregnancy		Birth		Immediate puerperium		Post partum		4 to 6 weeks		Month 3			Month 4		Month 6
Ad hoc questionnaires (Research Electronic Data Capture)																		
Informed consent to be signed			✓															
General sociodemographic and clinical data			✓															
**Newborn variables**					✓		✓		✓		✓							
	Breastfeeding observation: Latch, Audible Swallowing, Type of Nipple, Comfort, and Hold scale					✓		✓										
	Obstetric pain: visual analog scale					✓		✓										
	Tongue tie					✓		✓										
	Nipple wounds					✓		✓										
	Other puerperium variables			✓		✓		✓										
Feeding type							✓						✓			✓		✓
**Mother’s nutrition variables**																		
	Purchasing and cooking habits									✓								
	The mother’s daily diet recall (24 h)									✓								
Feeding accessories									✓

**Data collection variables.**

**Prenatal primary care data**

- Study recruitment survey: confirmation of informed consent and inclusion criteria
- Sociodemographic and clinical data

**Hospital data at the time of delivery**

- Delivery room survey: birth and breastfeeding initiation data
- Maternity department survey: data from the visual analog scale (VAS) [31]; assessment of breastfeeding, such as the Latch, Audible Swallowing, Type of Nipple, Comfort, and Hold (LATCH) scale [32]; refer to Multimedia Appendix 1 for ankyloglossia and other variables

**Postpartum data (primary care data)**

- Early postpartum survey (primary care; 1-2 weeks after birth): data from the VAS [31] and breastfeeding assessment, such as the LATCH scale [32], ankyloglossia, etc (Multimedia Appendix 1)
- Quarantine survey (4-6 weeks after birth): postpartum survey that includes data from the VAS [25] and breastfeeding assessment, such as the LATCH scale [31] and frenulum
- Survey on infant feeding and accessories
- Survey on the nutrition of the mother
- Ferrari 24-hour recall of maternal diet [33,34]
- Eating and cooking habits
- Phone call survey after 6 months: survey on types of infant feeding from 2 to 6 months; survey to evaluate the use of educational and support interventions
- Survey on withdrawal from the study

### Data Analysis Plan

The effectiveness of the educational intervention will be evaluated as differences between the EG and CG across 3 principal outcome variables. These outcomes are derived from the data collected during the study and are structured from general descriptive indicators to specific interventional impacts.

#### Infant Feeding Practices

Breastfeeding practices will be assessed from birth to 6 months of age. The incidence and prevalence of EBF, mixed feeding, and formula feeding will be calculated.

#### Maternal Nutrition

Maternal dietary intake will be evaluated in comparison with the recommended nutritional standards [35], focusing on caloric intake (total kilocalories consumed), macronutrient distribution (proportions of carbohydrates, proteins, and fats [saturated, monounsaturated, and polyunsaturated]), and micronutrient levels (iron, calcium, magnesium, and vitamin D).

The analysis will determine whether the average maternal diet meets the recommended nutritional guidelines.

#### Environmental Impact Assessment

Environmental impacts associated with infant feeding practices and maternal diet will be assessed at 6 weeks post partum. This includes consumption of artificial milk, use of feeding accessories, and maternal dietary habits.

A comparative life cycle assessment will be conducted in accordance with ISO 14040:2006, focusing on 3 environmental indicators:

- Climate change—measured in kg CO2 equivalents using the Intergovernmental Panel on Climate Change Global Warming Potential 100-year method [36]
- Water consumption—measured in m3 using the ReCiPE method, accounting for both direct and indirect water use [37]
- Water scarcity—measured in m3 equivalents using the available water remaining method [38]

### Statistical Analysis

Categorical variables (such as gender, country of birth, and infant feeding type) will be summarized using absolute and relative frequencies for the total and categories.

Quantitative variables will be described using means and SDs for normally distributed data and medians and 95% CIs for nonnormally distributed data.

The differences will be checked with the chi-square test for categorical variables and ANOVA for continuous variables with normal distribution or the Kruskal-Wallis *H* test for continuous variables with nonnormal distribution. The statistical significance will be set at *P*<.05. The registers with missing data for main outcome variables will not be included in the analysis.

## Results

### Progress Timing and Flow of Participants Through the Study

The timeline and implementation status of phase 2 of the Green Mother project are mentioned subsequently.

#### Implementation Status

Following approval by the ethics committee on February 22, 2023, the research team organized a multidisciplinary group of experts to develop the *Guide to Good Practices in Breastfeeding, Nutrition, and Sustainability*. This guide was based on the Clinical Practice Guide on Breastfeeding published by the Spanish Ministry of Health and was developed during the first half of 2023.

#### Guide Development and Professional Training

A total of 16 health care professionals, each specialized in relevant content areas, contributed to the development of the guide. In addition, a 20-hour training course was organized to educate professionals on best practices in breastfeeding, nutrition, and environmental impact. This training was conducted between June 2023 and July 2023.

#### Recruitment Status

Recruitment of pregnant women began in September 2023 and finished in December 2024.

#### Data Collection and Follow-Up

Data collection was conducted through follow-up of participants until 6 months post partum for the last recruited woman. This phase extended until September 2025, with complementary data collection and revision in October 2025.

#### Data Exploitation and Analysis Status

Between November 2025 and January 2026, the research team will complete data cleaning and afterward conduct the final data analysis. The final results are anticipated to be ready for publication and dissemination in 2026.

**Figure 2:**
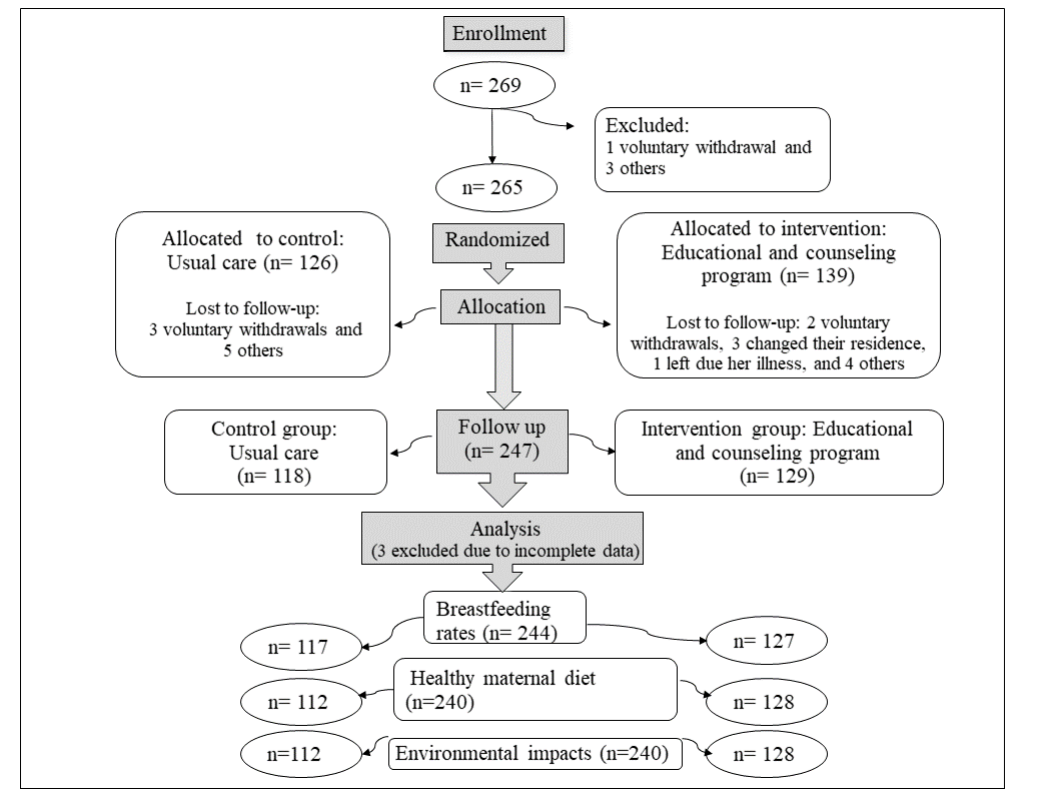
Flow chart of study participation.

The participant flowchart is presented in Figure 2.

### Citizen Participation

Mothers involved in breastfeeding and parenting will collaborate with the research team to evaluate and validate the content of informational pamphlets and other resources of the proposed educational interventions before implementation. Their opinions will also be considered for discussion of the project results and their subsequent dissemination to communities and the public.

## Discussion

### Expected Results

Starting with the objective of evaluating the effectiveness of this educational and reinforcing intervention, we expect to find the following differences between the 2 groups:

- A higher rate of EBF among EG mothers in the first 6 months of the baby’s life
- A lower environmental impact associated with infant feeding, considering the use of accessories, the mothers’ diet, and eating habits among mothers in the EG at 1 month of the baby’s life
- A more healthful diet and a more adequate proportion of nutrients in the mothers’ diets in the EG

It is crucial to adopt evidence-based strategies and new guidelines for complementary education in aspects such as breastfeeding, mothers’ nutrition, and environmental information for the health personnel involved in the care of pregnant and breastfeeding women.

Group education, both written and audiovisual dissemination, has proven to be an effective tool used by primary care professionals. The self-determination of women and their partners as providers of food for the entire family and their role in obtaining sustainable food must be reinforced.

Once the project is completed, the guide, different materials, and courses will be used to train all health professionals in the entire territory of Catalonia involved in mother and baby care to focus on the nursing mother, providing the best conditions for a good start to breastfeeding and offering support for any difficulties that may arise. It will also include information on how to follow a healthy and environmentally friendly diet.

### Limitations and Strengths

Prenatal counseling and education in primary care centers will be updated with the materials created for this project, and they will be disseminated to all health centers. Potential limitations of the study during data collection are that participants in the CG, without specific intervention, can obtain information through social networks, television, and the internet. However, to control for this fact, we included a final questionnaire to verify the sources of information.

In this study, all participants intending to breastfeed are included, as its objective is not to influence their decision of which type of infant feeding, they choose but rather to support those who wish to breastfeed for an extended period (6 months), helping them achieve this successfully and without complications. For the infant feeding rates in the general case, we conducted phase 1 of the Green Mother project, and from the previous study, LACTEM [21], from the same geographic area, we found that 96% of the pregnant mothers wanted to breastfeed. The mothers’ illness, country of birth, and age were also observed as facilitators of and barriers to breastfeeding. During the recruitment phase 1 of this project, which investigated the environmental impact of different types of infant feeding, we observed a 93% breastfeeding rate at hospital discharge [12].

Furthermore, all users with the intention of breastfeeding are included because the objective of this study is not to influence the type of breastfeeding that the participants will choose but rather to ensure that those who want to breastfeed for a long time can achieve it. This also helps to see the rates of those who wanted but could not initiate or follow the EBF or breastfeeding.

The development of this research will not only enable the updating of standard prenatal programs but also contribute to the achievement of the Sustainable Development Goals (SDGs), particularly those related to health (SDG 3), gender equality (SDG 5), responsible consumption (SDG 12), and climate action (SDG 13). Ultimately, strengthening health education through an environmental lens may foster a shift in attitudes toward healthier and more sustainable practices, thereby ensuring the long-term well-being of both the population and the planet [25,26].

### Conclusions

The results of this study will demonstrate the effectiveness of the educational and counseling intervention. This intervention will not only help improve support for prolonged breastfeeding and therefore benefit children’s health but also help reduce environmental impact, raising awareness among mothers and families of the importance of breastfeeding their children and having healthy and sustainable diets, as stated in the 2030 SDGs.

The findings of this project will be shared with both the scientific community and the public through various channels. These include continuing education courses, informative talks, community discussions, as well as presentations at conferences and congresses. By disseminating the results through these media, the knowledge gained from the project will reach a broader audience and contribute to the advancement of understanding in the field and the achievement of the SDGs.

## Appendix

Multimedia Appendix 1 Study surveys.

Multimedia Appendix 2 CONSORT 2025 checklist.

Multimedia Appendix 3 Peer review report from the PERIS Primary Care Programme, Agency for Health Quality and Assessment of Catalonia (AQuAS; Spain).

## Abbreviations

ASSIR: Atenció a la Salut Sexual i Reproductiva (reproductive and sexual health care unit)
CG: control group
EBF: exclusive breastfeeding
EG: experimental group
GHG: greenhouse gas
REDCap: Research Electronic Data Capture
SDG: Sustainable Development Goal
WHO: World Health Organization

## Data Availability

Data sharing is not applicable to this study as no datasets were generated or analyzed during this study.

## References

[1] Joffe N, Webster F, Shenker N (2019). Support for breastfeeding is an environmental imperative. BMJ.

[2] Legendre M, Pirozzi G Convención Sobre Los Derechos Del Niño. UNICEF.

[3] Pérez-Escamilla R, Tomori C, Hernández-Cordero S, Baker P, Barros A, Bégin F, Chapman DJ, Grummer-Strawn LM, McCoy D, Menon P, Ribeiro Neves PA, Piwoz E, Rollins N, Victora CG, Richter L, 2023 Lancet Breastfeeding Series Group (2023). Breastfeeding: crucially important, but increasingly challenged in a market-driven world. Lancet.

[4] (2010). La alimentación del lactante y del niño pequeño. World Health Organization (WHO).

[5] Kramer MS, Aboud F, Mironova E, Vanilovich I, Platt RW, Matush L, Igumnov S, Fombonne E, Bogdanovich N, Ducruet T, Collet JP, Chalmers B, Hodnett E, Davidovsky S, Skugarevsky O, Trofimovich O, Kozlova L, Shapiro S, Promotion of Breastfeeding Intervention Trial (PROBIT) Study Group (2008). Breastfeeding and child cognitive development: new evidence from a large randomized trial. Arch Gen Psychiatry.

[6] Sayres S, Visentin L (2018). Breastfeeding: uncovering barriers and offering solutions. Curr Opin Pediatr.

[7] Karlsson JO, Garnett T, Rollins NC, Röös E (2019). The carbon footprint of breastmilk substitutes in comparison with breastfeeding. J Clean Prod.

[8] Andresen EC, Hjelkrem AG, Bakken AK, Andersen LF (2022). Environmental impact of feeding with infant formula in comparison with breastfeeding. Int J Environ Res Public Health.

[9] Cos-Busquets J, Cabedo-Ferreiro RM, Liutsko L, Reyes-Lacalle A, García-Sierra R, Colldeforns-Vidal M, Andrade EP, Vicente-Hernández MM, Gómez-Masvidal M, Montero-Pons L, Torán-Monserrat P, Falguera-Puig G, Cazorla-Ortiz G, GREEN MOTHER Group (2025). A comprehensive assessment of the environmental impact of different infant feeding types: the observational study GREEN MOTHER. J Adv Nurs.

[10] Serra-Majem L, Tomaino L, Dernini S, Berry EM, Lairon D, Ngo de la Cruz J, Bach-Faig A, Donini LM, Medina FX, Belahsen R, Piscopo S, Capone R, Aranceta-Bartrina J, La Vecchia C, Trichopoulou A (2020). Updating the mediterranean diet pyramid towards sustainability: focus on environmental concerns. Int J Environ Res Public Health.

[11] Alimentació durant l'embaràs. Gencat.

[12] Reyes-Lacalle A, Cabedo-Ferreiro RM, Cos-Busquets J, Liutsko L, Colldeforns-Vidal M, García-Sierra R, Vicente-Hernández MM, Gómez-Masvidal M, Montero-Pons L, López-Gimeno E, Torán-Monserrat P, Falguera-Puig G, Cazorla-Ortiz G (2025). Characteristics, preventive factors, and barriers to breastfeeding and mixed feeding in the first month of life in Barcelona: the multicenter observational study GREEN MOTHER. Nutrients.

[13] (2019). Green feeding-climate action from birth. International Baby Food Action Network.

[14] Martinelli SS, Cavalli SB (2019). Healthy and sustainable diet: a narrative review of the challenges and perspectives. Cien Saude Colet.

[15] The Paris agreement. United Nations.

[16] Metas mundiales de nutrición 2025: documento normativo sobre lactancia materna. Organizasión Mundial de la Salud.

[17] Brown A (2017). Breastfeeding as a public health responsibility: a review of the evidence. J Hum Nutr Diet.

[18] Rana R, McGrath M, Sharma E, Gupta P, Kerac M (2021). Effectiveness of breastfeeding support packages in low- and middle-income countries for infants under six months: a systematic review. Nutrients.

[19] Shafaei FS, Mirghafourvand M, Havizari S (2020). The effect of prenatal counseling on breastfeeding self-efficacy and frequency of breastfeeding problems in mothers with previous unsuccessful breastfeeding: a randomized controlled clinical trial. BMC Womens Health.

[20] Souza ED, Pina-Oliveira AA, Shimo AK (2020). Effect of a breastfeeding educational intervention: a randomized controlled trial. Rev Lat Am Enfermagem.

[21] Cabedo R, Manresa JM, Cambredó MV, Montero L, Reyes A, Gol R (2019). Types of breastfeeding and factors that influence its abandonment up to 6 months. LACTEM Study. Matronas Profesión.

[22] Sattari M, Serwint JR, Levine DM (2019). Maternal implications of breastfeeding: a review for the internist. Am J Med.

[23] Ota E, Hori H, Mori R, Tobe-Gai R, Farrar D (2015). Antenatal dietary education and supplementation to increase energy and protein intake. Cochrane Database Syst Rev.

[24] Jadotte YT, Caron RM, Kearney GD (2022). Ecosystemic theory, practice, and policy: training recommendations for environmental public health. Am J Prev Med.

[25] Liutsko L (2019). The integrative model of personality and the role of personality in a planetary health context. Pers Individ Differ.

[26] Lerner H, Berg C (2017). A comparison of three holistic approaches to health: one health, ecohealth, and planetary health. Front Vet Sci.

[27] Cabedo-Ferreiro RM, Liutsko L, Cos-Busquets J, García-Sierra R, Colldeforns-Vidal M, Reyes-Lacalle A, Vicente-Hernández MM, Gómez-Masvidal M, Montero-Pons L, Cazorla-Ortiz G, Torán-Monserrat P, Violán C, Falguera-Puig G, GREEN MOTHER Group (2024). Environmental impact of infant feeding type, accessories used and maternal dietary habits: the GREEN MOTHER-I project, a cross-sectional study protocol. Nutr J.

[28] Reyes-Lacalle A, Montero-Pons L, Manresa-Domínguez JM, Cabedo-Ferreiro R, Seguranyes G, Falguera-Puig G (2020). Perinatal contraceptive counselling: effectiveness of a reinforcement intervention on top of standard clinical practice. Midwifery.

[29] Jané M, Amorós P, Molina MC, Mateum A Pla de salut afectiva i sexual (PSAS). Agència de Salut Pública de Catalunya.

[30] Harris PA, Taylor R, Minor BL, Elliott V, Fernandez M, O'Neal L, McLeod L, Delacqua G, Delacqua F, Kirby J, Duda SN, REDCap Consortium (2019). The REDCap consortium: building an international community of software platform partners. J Biomed Inform.

[31] Scott J, Huskisson EC (1976). Graphic representation of pain. Pain.

[32] Báez León C, Blasco Contreras R, Martín Sequeros E, Pozo Ayuso ML, Sánchez Conde AI, Vargas Hormigos C (2008). Validación al castellano de una escala de evaluación de la lactancia materna: el LATCH. Análisis de fiabilidad. Index Enferm.

[33] Cabedo R, Manresa JM, Cambredó MV, Montero L, Reyes A (2019). Tipos de lactancia materna y factores que influyen en su abandono hasta los 6 meses. Matronas Prof.

[34] Ferrari MA (2013). Estimación de la Ingesta por Recordatorio de 24 Horas. Diaeta.

[35] European Food Safety Authority (EFSA) (2017). Dietary reference values for nutrients summary report. EFS3.

[36] Kirschbaum MU (2014). Climate-change impact potentials as an alternative to global warming potentials. Environ Res Lett.

[37] Dekker E, Zijp MC, van de Kamp ME, Temme E, van Zelm R (2019). A taste of the new ReCiPe for life cycle assessment: consequences of the updated impact assessment method on food product LCAs. Int J Life Cycle Assess.

[38] Boulay AM, Bare J, Benini L, Berger M, Lathuillière M, Manzardo A, Margni M, Motoshita M, Núñez M, Pastor AV, Ridoutt B, Oki T, Worbe S, Pfister S (2017). The WULCA consensus characterization model for water scarcity footprints: assessing impacts of water consumption based on available water remaining (AWARE). Int J Life Cycle Assess.

