# Supplementary material for: Effectiveness of an Educational and Counseling Program (the Green Mother Project Phase 2) to Enhance Breastfeeding and Improve Mothers’ Diets From an Environmental Perspective: Protocol for a Cluster Randomized Controlled Trial
Source: JMIR Res Protoc. 2026 Jan 26;15:e80358. doi: 10.2196/80358 (PMC12887563; doi:10.2196/80358)
Supplement: Multimedia Appendix 1 [file resprot_v15i1e80358_app1.docx]

**Appendix I. The Study Surveys**

1. *Sociodemographic and clinical profile*:

• Age

• Gender: female, male, non-binary, other

• Relationship status: male partner, female partner, non-binary partner, no partner, other

• Region of birth: Spain, Rest of Europe, North Africa, Sub Saharan Africa, Asia, Hindustan region, Central and South America, North America, Oceania

• Level of studies: without studies, primary school, secondary studies, university studies

• Religion: Islam, Hindu, Buddhist, Christian, Jewish, agnostic, others

• Paid work: yes/no

• Multiple pregnancy: yes/no

• TPAL: N of Term births, Preterm births, Abortions, Living children

• Baby’s birth weight: in grams

• Weeks of gestation at birth: weeks

• Maternity leave: yes/no, expected duration in weeks

• Partner’ leave to care for the baby: yes/no, expected duration in weeks

• Maternal pathology: insulin-induced diabetes, type II diabetes, hypertension, digestive disease, immunological disease, endocrine disease, mental illness, breast surgery, and others

• Neonatal pathology: low birth weight baby, premature, jaundice/high bilirubin, newborn hospitalization

2. *Hospital data on mothers*:

a. Birth (delivery room)

• Baby’s birth weight:

• Weeks of gestation at birth:

• Type of delivery: eutocic/instrumented/caesarean section

• Resuscitation of newborn: yes/No

• Skin to skin: 0-30 min, 30-60 min, 60-120 min

• Start of BF: 0-30 min, 30-60 min, 60-120 min., >120 min

• Spontaneous grasping of the nipple: yes/no

• Separation of mother and baby: yes/no

3. *Immediate puerperium (hospital discharge)*

• Length of stay in hospital: days

• Nipple wounds: yes/no

• Engorgement: yes/no

• Diagnosed tongue tie: yes/no (T1- birth/T2 - postpartum)

Type of tongue tie: I-II-III-IV (T1/T2)

Frenectomy: yes/no (T1/T2)

• Type of feeding in hospital: EBF, MF, FF

• Obstetric pain, VAS Scale: analogue 0-10 and with faces

• Baby’s weight: first day of life; weight loss in grams (< 7%, 7-10%, > 10%)

• Observation LATCH Scale: yes/no 0-10

• Number of feedings per day

4. *One week postpartum (primary health)*

• Nipple wounds: yes/no

• Engorgement: yes/no

• Diagnosed tongue tie: yes/no (T1- birth/T2 - postpartum)

• Obstetric pain, VAS Scale: analogue 0-10 and with faces

• Baby’s weight: first day of life; weight loss in grams (< 7%, 7-10%, > 10%)

• Baby weight gain at 1 week of life (in grams)

• Observation LATCH Scale: yes/no 0-10

• Feeding type: EBF, MF, FF

• Number of BF feedings per day

• Number of FF feedings per day

5. *Postpartum quarantine control*

 • Did you have family help? Yes/no

 • Participation in the maternal (paternal) education group: yes/no

• Participation in the postpartum BF support group: yes/no

• Feeding type: EBF, MF, FF

• Nipple wounds: yes/no

• Engorgement: yes/no

• Diagnosed tongue tie: yes/no (T1- birth/T2 - postpartum)

• Obstetric pain, VAS Scale: analogue 0-10 and with faces

• In the case of MF or FF, indicate how much infant formula you feed your baby daily, in ml:

• Number of bottles or syringes of infant formula you give your baby daily: from 1 to more than 8 ~~par~~per day

• How many ml does each bottle or syringe have: 10-150 ml

• How much water is used to prepare the infant formula: ml/day

6. *Feeding accessories:*

• Do you use a breast pump: yes/no

• Do you use milk storage containers: yes/no

• Total number of milk storage containers you own:

• Do you use bags to store milk: yes/no

• How many bags do you use each week:

• Do you use nipple shields: yes/no

• Do you use a nursing supplementer: yes/no

• How many cans of infant formula do you use weekly: yes/no and number per week (based on an 800 gr can)

• Number of containers/bottles you have at home:

• Do you have bottles: yes/no, and number

• Do you use brushes to clean the bottles: yes/no

• Do you use a bottle warmer: yes/no

• Do you use a bottle sterilizer: yes/no

• Do you use paediatric nasogastric tubes: yes/no

• Reusable nursing pads: yes/no

• Disposable nursing pads: yes/no, and how many do you use per week.

7. *Mother's nutrition variables (purchasing and cooking habits)*

• What type of diet do you follow: no special diet, vegetarian, vegan, gluten-free, food allergies, therapeutic diet (diabetes, high blood pressure, dyslipidemia, etc.)

• Do you take any vitamin supplements: yes/no

• Where do you usually buy food: supermarket, take away, small business (neighborhood, market), local product (km 0) usually, local product (km 0) less than 50%, local product (km 0) more than 50%

• How do you usually go shopping: car: 0-5 km, Car: 6-10 km, Car: > 11 km, walking, public transport

• Type of energy in your kitchen: butane gas, natural gas, electricity, biomass (wood)

• Members of the family who usually cook: mother, partner, both, others

• How much of the food do you end up throwing away: from < 5%, 6-10%, 11-24%, > 25%

8. *Mother's nutrition variables (mother's diet recall (24h))*

• Diet. “24-hour food recall. 24hR”: three main meals and snacks (before breakfast, mid-morning, afternoon, and before bed)

• Type of food: portions, packaging, type of cooking, characteristics of the product (fresh, frozen), origin of the product, condiments and drink

• Packaging: glass, Tetra Brik, plastic, paper, no packaging

• Characteristics of the food: fresh, frozen, pre-cooked, canned, preserved in glass

• Cooking methods: raw, boiled, steamed, fried, baked, or grilled

• Sauces and condiments: mayonnaise, ketchup, tomato, mustard, soy, oil, vinegar, butter

• Beverages: water, carbonated soft drinks (Coca-Cola, lemonade, etc.), packaged juices, alcoholic beverages, vegetable milk or animal milk

• Food measurement: small (100 gr), medium (200 gr), large (300 gr); fruit: ½ piece small portion, 1 piece (medium portion), 2 large pieces.

9. *Phone call survey 6 months:*

• Have you had any information about the benefits of breastfeeding

• Have you had any information about healthy diet

• Have you had any information about sustainable diet

• You have used BF spaces or resources

• You have participated in some postpartum or breastfeeding support space

• You have had individual advice from the professional

• Type of breastfeeding at 2 months of life: EBF. MF. FF

• Type of breastfeeding at 3 months of life: EBF. MF. FF

• Type of breastfeeding at 4 months of life: EBF. MF. FF

• Type of breastfeeding at 5 months of life: EBF. MF. FF

• Type of breastfeeding at 6 months of life: EBF. MF. FF

• The main cause of breastfeeding abandonment: mother's will, pain, mother's health, health of the baby, joining work, the feeling of not having enough milk, others
