# Supplementary material for: Effectiveness of an Educational and Counseling Program (the Green Mother Project Phase 2) to Enhance Breastfeeding and Improve Mothers’ Diets From an Environmental Perspective: Protocol for a Cluster Randomized Controlled Trial
Source: JMIR Res Protoc. 2026 Jan 26;15:e80358. doi: 10.2196/80358 (PMC12887563; doi:10.2196/80358)
Supplement: Multimedia Appendix 3 [file resprot_v15i1e80358_app3.pdf]

Evaluation Report

Evaluation Report

Call: PERIS - 5th Call (SLT021)
Instrumental Action: Grants for oriented research projects: primary care modality

File: SLT021/21/000063

Score:
• Scientific-technical proposal: 52.75
• Research team: 28.50
• TOTAL: 81.25

Evaluation Justification:
The study aims, in a first phase, to evaluate the environmental impact in terms of carbon footprint of breastfeeding vs. formula feeding. The footprint is mainly based on determining the necessary accessories and the waste generated by feeding in the first month of life. The second phase aims to evaluate the effectiveness of a standardized intervention on breastfeeding, diet, and environmental impact in the first month of life and how this intervention impacts breastfeeding in the first six months of life. The first phase will be conducted with a descriptive observational design. The second phase will be carried out with a quasi-experimental design with a non-equivalent control group. It is a multicenter study with different provider units. The environmental impact is focused solely on the first month of life, although the authors justify this due to the large volume of carbon dioxide produced in this first month and because there is a high prevalence of mothers who abandon breastfeeding after the first few months. Each month in a baby's life has special characteristics; for this reason, it would be very interesting to extend this follow-up over a longer breastfeeding process.

It is an innovative proposal that incorporates research on environmental impact in the healthcare process, in this case, breastfeeding. It is a multicenter study with an intervention that aims not only to evaluate this footprint but also to reduce it through specific training. It is a highly valued and feasible proposal.
